# We Found a Gem in Your Heart: Valvular Heart Disease and Infective Endocarditis Discovered

**DOI:** 10.7759/cureus.42176

**Published:** 2023-07-20

**Authors:** Roy Lim, Justin Barimayena, Kelsie-Ann Mita, Brian Denney, Reejeen M Coz

**Affiliations:** 1 Internal Medicine, Mount Sinai Hospital, Chicago, USA; 2 Pharmacology and Therapeutics, Roosevelt University College of Science, Health and Pharmacy, Schaumburg, USA; 3 General Medicine, Cebu Velez General Hospital, Cebu, PHL; 4 Internal Medicine, Our Lady of Fatima University, Valenzeula, PHL

**Keywords:** heart vegetations, gemella morbillorum, gemella endocarditis, ie, infective endocarditis, vhd, valvular heart disease, gemella

## Abstract

Valvular heart disease (VHD) occurs when there is a functional impairment in the valvular apparatus that either obstructs or regurgitates the backflow of blood. When a microorganism resides in those valves, it injures the leaflets and causes complications such as thromboembolic events. Infective endocarditis (IE), usually caused by the Staphylococci and Streptococcus group, is a disease that occurs on the heart valves. Antibiotic resistance is common; thus, culture and sensitivity testing should be done for a more targeted treatment approach. We herein present a rare case of *Gemella morbillorum* (*G. morbillorum*) vegetations found in a patient's heart that initially presented with cerebrovascular disease symptoms and underwent heart surgery in the end.

## Introduction

Valvular heart disease (VHD) is a condition with defective valve leaflet/s whose sole function is to provide unobstructed forward blood flow throughout the heart [1]. This condition can be congenital, secondary to inflammation, or a complication of an infection. Valvular damage or malfunction can result in either stenosis or regurgitation. Stenosis occurs when a valve fails to open properly, which results in limited blood flow. On the other hand, regurgitation occurs when the valves do not close firmly, which allows blood to leak back to the proximal chambers [1]. Hence, there should be unhampered blood flow to prevent significant medical problems resulting in shortness of breath or dyspnea, palpitations, reduced functional capacity, and worse fulminant heart failure.

*Gemella morbillorum* (*G. morbillorum*) infective endocarditis (IE) is a rare condition that brings about significant morbidity [2]. The morbidity and mortality rates of *G. morbillorum* endocarditis are still high despite the developments in treatment methods. Any delays in treatment can predispose to embolic or immunological events [3,4]. One of the most common potentially disabling embolic events of IE is stroke [5]. It results from the embolization of valvular vegetations that subsequently occludes cerebral circulation [6].

Due to its rarity, there has been limited literature on *Gemella*, with most of it consisting of case reports [2]. This paper describes a case of *G. morbillorum *IE alongside VHD. The patient initially presented with left lower extremity weakness. On workup, a CT scan plain of the brain showed ill-defined hypodensity in the left pontomedullary junction. MRI showed a moderate infarct in the right medial, frontal, parietal, and pericallosal regions. Transesophageal echocardiogram (TEE) showed bicuspid aortic valve, severe aortic regurgitation (AR), moderate mitral regurgitation (MR), moderate to severe tricuspid regurgitation (TR), and aortic and mitral valve vegetation. Blood culture showed *G. morbillorum*. The patient underwent open heart surgery with aortic valve replacement, mitral valve replacement, and tricuspid valve annuloplasty.

## Case presentation

The case involves a 40-year-old male who presented to the emergency department with sudden onset left leg weakness and heaviness while at work. The patient is a Spanish speaker with no known past medical history. He denied experiencing fever, chills, headache, dizziness, nausea, vomiting, chest pain, shortness of breath, abdominal pain, leg swelling, or urinary or bowel symptoms. The patient reported undergoing a wisdom tooth extraction two years prior but otherwise had no significant medical history. He also denied any history of hypertension, heart conditions, or illicit drug use. Vital signs were generally within normal limits, except for a blood pressure of 154/54 mmHg and a heart rate of 115 beats per minute. On physical examination, the patient was found to have a diastolic murmur in the left lower sternal border and decreased strength (4/5) in the left lower extremity.

**Figure 1:**
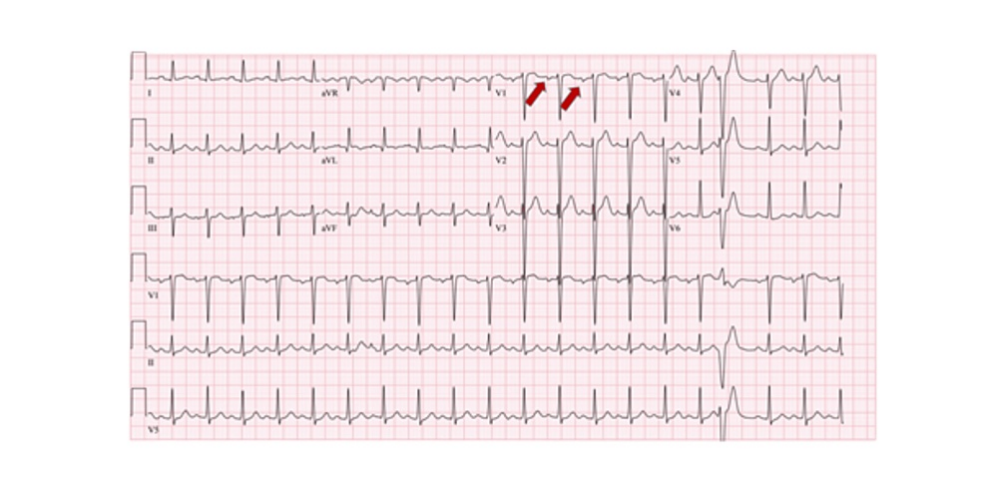
ECG showing sinus tachycardia and left atrial enlargement (red arrow)

Figure 1 shows the ECG of the patient, which revealed sinus tachycardia with occasional premature ventricular complexes.

**Figure 2:**
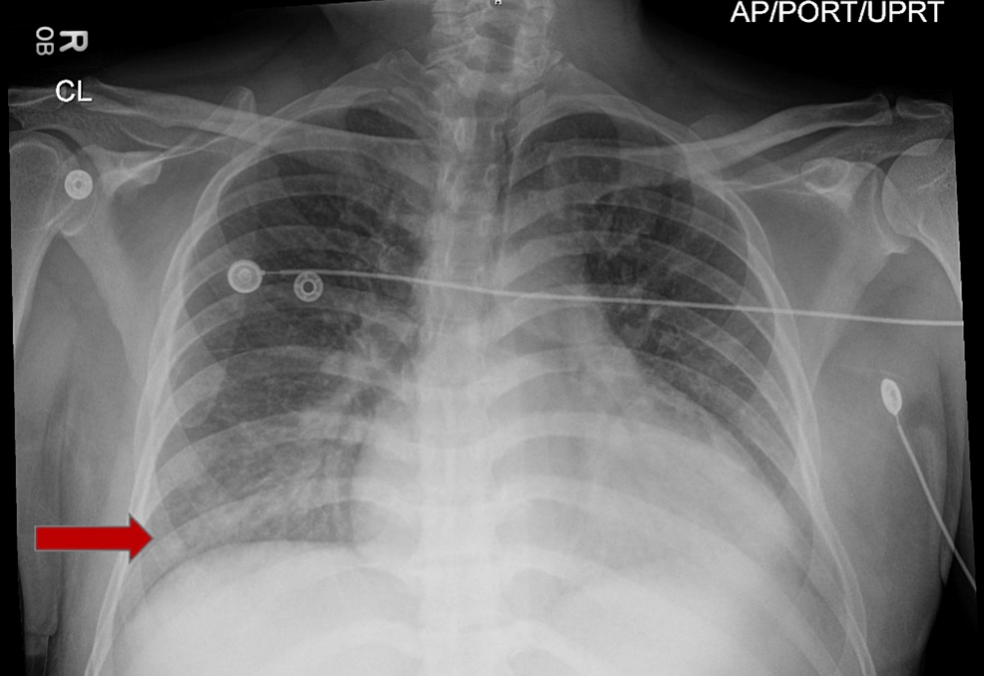
Chest X-ray showing pulmonary congestion and new ground-glass opacity in the left lower lung zone (red arrow)

The chest X-ray, as illustrated in Figure 2, showed evidence of mild pulmonary congestion and basilar atelectasis. Additionally, a new ground-glass opacity was identified throughout the left lower lung zone.

The blood work results presented in Table 1 indicate mild leukocytosis with a WBC count of 12.4, mild anemia with a hemoglobin level of 11.6, mildly elevated troponin I HS of 21, and a B-type natriuretic peptide level of 537.

**Table 1:** Laboratory workup results on admission which displays the patient's blood work results CO2: carbon dioxide, BUN: blood urea nitrogen, BNP: B-type natriuretic peptide, WBC: white blood cell, RBC: red blood cell, *Hgb: hemoglobin, **Hct: hematocrit

Test	Result	Reference value
Sodium	134	133-144 meq/L
Potassium	3.8	3.5-5.2 meq/L
Chloride	101	98-107 meq/L
Calcium	8.7	8.6-10.3 mg/dL
CO_2_	23	21.0-31.0 meq/L
Anion gap	10	4-11 mmol/L
Glucose	143	70-99 mg/dL
BUN	21	7-25 mg/dL
Creatinine	1.1	0.7-1.3 mg/dL
BUN/creatinine ration	19	5-19
Troponin I	21	0-0.04 ng/mL
BNP	531	<100 pg/mL
WBC count	12.4	4.0-11.0 x 10^3^/uL
RBC count	4.44	4.34-5.60 x 10^6^/uL
Platelet count	241	150-450 x 10^3^/uL
*Hgb	11.6	13.5-17.5 g/dL
**Hct	35.5	38.6-49.2%

**Figure 3:**
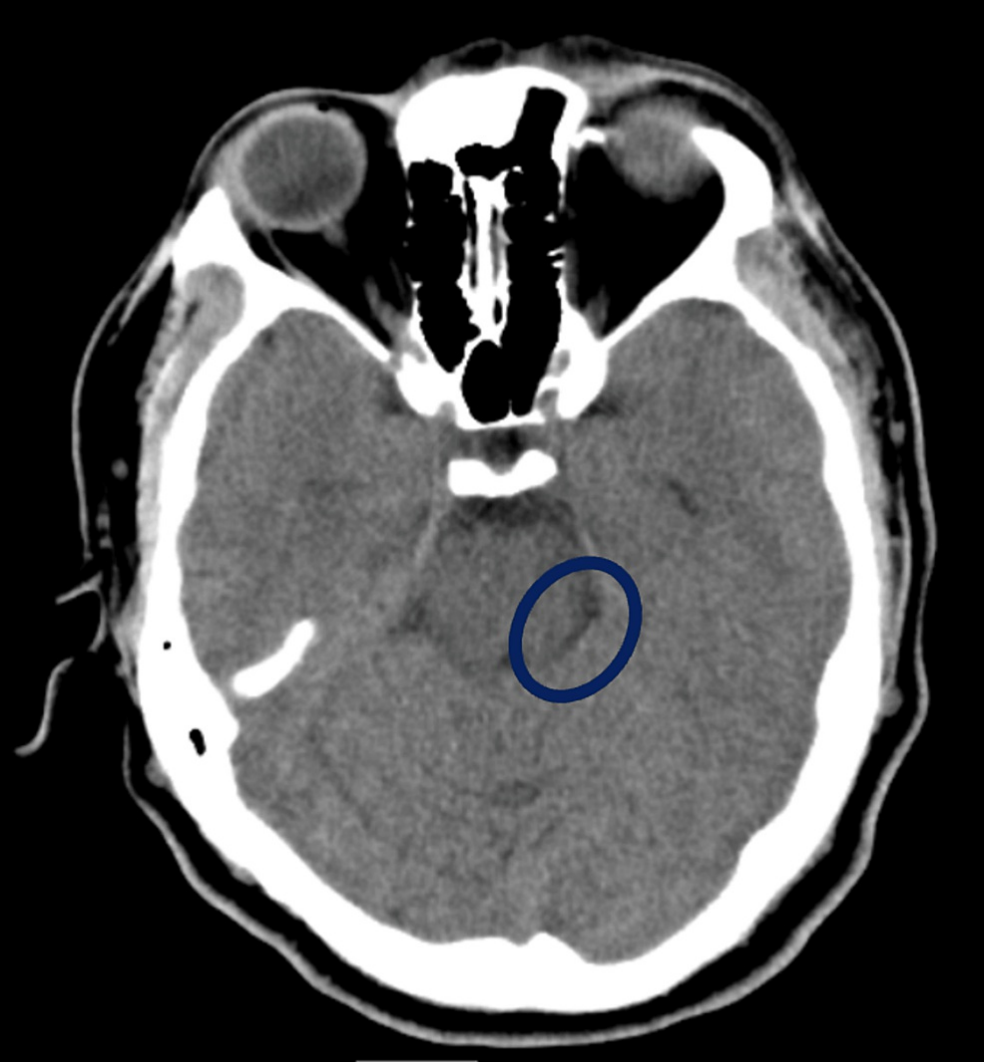
CT brain showing an ill-defined hypodensity in the left pontomedullary junction (blue circle)

The patient's left leg weakness prompted a CT scan of the brain, with results presented in Figure 3. Notably, the scan revealed an ill-defined hypodensity in the left pontomedullary junction, suggesting a possible lesion in that area. To further investigate, CT angiography of the head and neck was performed, which did not show any definitive large vessel occlusion.

The patient received a tissue plasminogen activator (TPA), and aspirin and statin medications were initiated. The permissive hypertension protocol was also applied. Following TPA administration, a repeat CT brain was performed, which demonstrated no significant interval changes from the prior imaging and no acute intracranial hemorrhage.

**Figure 4:**
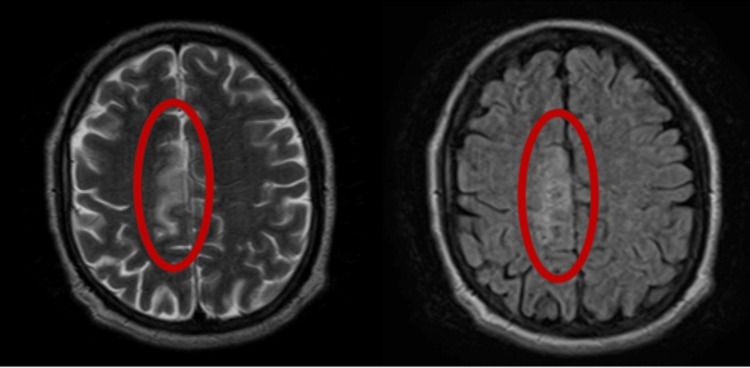
Brain MRI (right and left red circles) showed moderate-sized infarct in the right medial frontal parietal pericallosal region, concerning for right anterior cerebral artery territory infarct; no hemorrhage was observed

Further diagnostic tests were conducted using brain MRI, and the results, presented in Figure 4, revealed a moderate-sized infarct in the right medial frontal parietal pericallosal region. The findings suggest a possible right anterior cerebral artery territory infarct, but no signs of hemorrhage were found.

**Figure 5:**
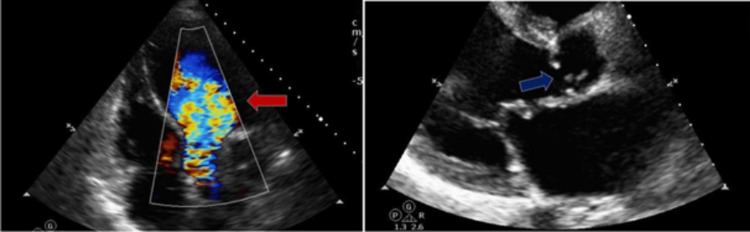
TTE with apical 5 chamber view (left) indicating the AR jet (red arrow); parasternal long axis view indicating the malcoaptation of the aortic valve leaflets (blue arrow)

**Figure 6:**
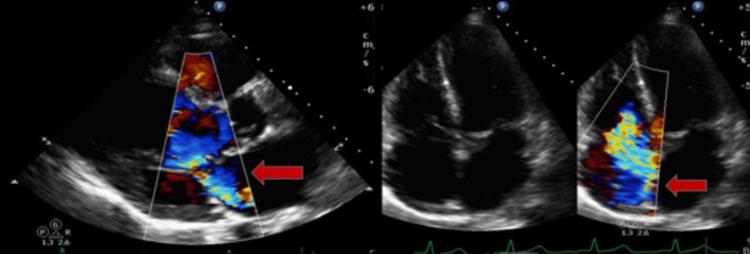
TTE with parasternal long axis view (left) showing moderate mitral valve regurgitation jet (red arrow); apical 4 chamber view (right) showing moderate to severe TR

The transthoracic echocardiogram (TTE) indicated a normal ejection fraction of 55-60% (Figures 5, 6). However, there was severe AR observed, with malcoaptation of the valve leaflets. Additionally, moderate MR and moderate-severe TR were present.

**Figure 7:**
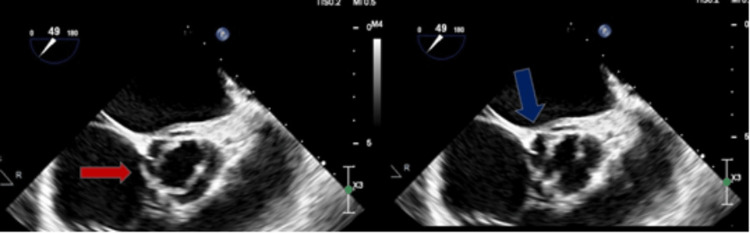
TEE with a midesophageal aortic valve long axis view (left) indicating bicuspid aortic valve (red arrow) and with the same view (right) showing the vegetation on the noncoronary cusp

**Figure 8:**
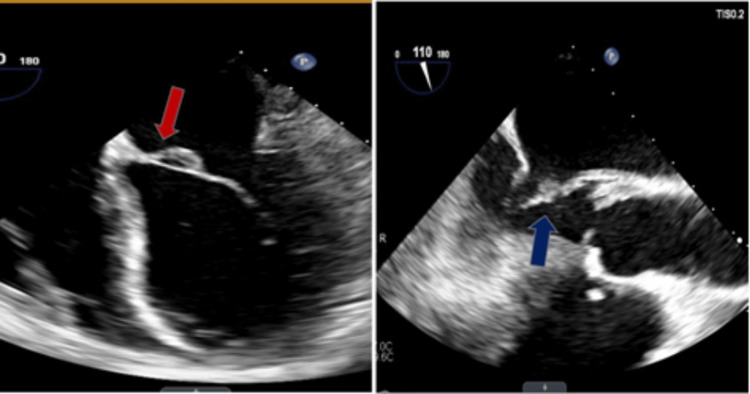
TEE with four-chamber view (left) indicating the vegetation on the anterior leaflet of the mitral valve (red arrow) and with midesophageal aortic long axis view (right) showing the same vegetation on the anterior leaflet of the mitral valve

Upon further investigation with a TEE (Figures 7, 8), it was discovered that the patient has a bicuspid aortic valve with a fusion of the right and left coronary cusps. Additionally, mobile vegetation measuring 0.8 cm and 0.4 cm was found on the noncoronary cusp, and a 0.4 cm x 0.3 cm vegetation was observed on the anterior leaflet of the mitral valve which likely seeded from the aortic valve with possible perforation.

Vancomycin and ceftriaxone antibiotics were started empirically. Subsequent blood culture results confirmed *G. morbillorum*, prompting consultation with the infectious disease team to determine the appropriate treatment. Following a discussion with the infectious disease team, it was determined that vancomycin was no longer necessary, and the patient’s treatment plan was modified accordingly.

Given the severity of the condition, the cardiothoracic surgery team was consulted and determined that open-heart surgery was the best course of action. The procedure involved replacing the aortic valve and mitral valve and performing a tricuspid valve annuloplasty. Fortunately, the surgery was successful, and the patient’s condition improved. After the surgery, the patient was then discharged to a rehabilitation center, where he received ongoing antibiotic therapy to ensure a complete recovery.

## Discussion

IE is a rare bacterial or fungal infection that affects the endocardium, the inner lining of the heart chambers or valves. The annual incidence of this condition is estimated to be between three and seven per 100,000 person-years [7]. Over the years, there has been a shift in the leading cause of IE from Streptococcus spp., commonly attributed to dental procedures in the past, to *Staphylococcus aureus* [7]. While *G. morbillorum*, a subspecies of viridans streptococci, is a known cause of endocarditis, its incidence in the literature is rare, with less than 40 documented cases [8]. Endocarditis can involve multiple heart valves, as evidenced by two case reports that documented multivalvular involvement of three valves. In one of these cases, surgery was required. The morbidity and mortality rates for endocarditis are estimated to be between 18% and 22%, which may be partly attributed to the limited data on optimal diagnosis, antibiotic stewardship, and surgical intervention for specific cases of *Viridans streptococcus* spp. [7]. The case presented here is that of a young, asymptomatic, and otherwise healthy male surgical candidate diagnosed with multivalvular *G. morbillorum* IE. The rarity of this presentation adds to the significance of this documented case, which provides valuable insights into the diagnosis and management of endocarditis caused by this uncommon subspecies of viridans streptococci.

IE can be caused by a variety of pathogens, including *Viridans streptococcus*, HACEK group, *Staphylococcus aureus*, community-acquired enterococci, and *Coxiella burnetii* [7]. The HACEK organisms include *Haemophilus* spp., *Aggregatibacter* spp., *Cardiobacterium hominis*, *Eikenella corrodens*, and *Kingella* spp. Among these, *Viridans streptococcus* is a complex and evolving group of organisms that can be difficult to grow, diagnose, and treat appropriately. This group encompasses several species, including *Streptococcus sanguinis*, *Streptococcus mitis*, *Streptococcus salivarius*, *Streptococcus mutans*, and *G. morbillorum*. The latter is a gram-positive coccus that is catalase-negative and a facultative anaerobe. It is a normal flora in the oropharynx and gastrointestinal and genitourinary tracts and accounts for less than 1% of cases of IE [9].

The development of IE begins with the introduction of pathogens into the bloodstream, leading to bacteremia and subsequent colonization of relatively sterile vegetations that adhere to native or prosthetic heart valves [7]. These vegetations typically consist of ball-like structures composed of fibrin and platelets, which provide potential substrates for bacteria in the bloodstream and possess an adhesive quality that can result in potentially fatal systemic complications [7].

The highest risk factors for patients presenting with pathogens like *G. morbillorum* are rheumatic disease and congenital heart disorders. The presence of implanted cardiac devices such as cardiac valve prostheses, implanted cardioverter-defibrillators, and permanent pacemakers increases the risk of infection [7]. Additionally, other risk factors include individuals with VHD, intravenous drug use, history of prior heart valve surgery, or history of previous IE [7]. If left untreated, IE can result in systemic complications, including strokes, seizures, paralysis, kidney damage, abscesses in the heart, lungs, or brain, as well as splenomegaly. *G. morbillorum* IE is associated with significant morbidity, with complications occurring in nearly two-thirds of cases, the most common being the need for cardiac surgery involving valve replacement. The antibiotic treatments recommended in the latest endocarditis guidelines have proven successful in treating the majority of cases [10].

IE typically presents with a constellation of signs and symptoms, including a new or changing heart murmur, aching joints and muscles, fatigue, fever, chills, night sweats, shortness of breath, and swelling in the feet or legs. Other less common symptoms may include Janeway lesions (painless red, purple, or brown flat spots on the soles or palms), Osler nodes (painful red or purple bumps or patches of darkened skin on the fingertips or toes), petechiae on the skin or whites of the eyes, or inside the mouth.

A definitive diagnosis of IE requires a combination of pathological and clinical criteria. According to the modified major Duke's Criteria, a microbiological assessment of three positive blood cultures is necessary, with the first and last cultures taken at least one hour apart. Additionally, an echocardiogram that shows vegetations on any of the heart valves is also required. CT and MRI are utilized to evaluate the spread of the disease to the brain, lungs, or other areas [7]. The goals of therapy for IE include early diagnosis, targeted antimicrobial therapy, and recognition of the need for early surgical intervention to improve patient survival [7].

The need for surgical intervention in cases of IE is determined by three clinical and echocardiographic features that encompass the vegetations, valvular dysfunction, and valve perforation or rupture. Echocardiography is utilized to evaluate the ejection fraction, which measures the percentage of blood that is pumped out of the heart. The TEE is preferred over the transthoracic approach for clearer imaging to identify vegetations on the heart valves. Surgery is required in some cases to prevent the risk of embolization. Pharmacologic therapy for *Viridans streptococcus* spp. is dependent on the minimum inhibitory concentration (MIC) for antibiotics targeted therapy, the type of valve (native vs. prosthetic), and the degree of susceptibility/resistance to penicillin. For native valve *Viridans streptococcus* IE susceptible to penicillin with a MIC of ≤ 0.12, the duration of antibiotic therapy is four weeks. Treatment options include aqueous crystalline penicillin G for four weeks, ceftriaxone for four weeks, penicillin or ceftriaxone for two weeks, ceftriaxone plus gentamicin for two weeks targeted at individuals with uncomplicated IE and healthy functioning kidneys, and intravenous vancomycin for four weeks for patients with type 1 IgE allergy or unable to tolerate penicillin or ceftriaxone therapy [7].

Regarding the case, the patient was a relatively young male patient who presented with stroke symptoms of left leg weakness and heaviness but with no signs of infection. The patient's dental history revealed wisdom tooth extraction performed two years ago, making it unlikely to be the source of infection. Upon detecting regurgitation on multiple valves in the initial TTE, further investigation was needed. The diagnosis was confirmed and vegetations were revealed by a subsequent TEE. Previous case reports have shown that in some instances, infections were caused by the retrograde flow of bacteria from the aortic valve, which subsequently seeded infections in other valves such as the mitral and tricuspid valves. While the literature on the optimal treatment for *G. morbillorum* is limited, studies on other *Viridans streptococcus* spp. suggest that penicillin plus gentamicin is the preferred treatment to achieve synergy. However, for this specific patient, a four-week course of ceftriaxone 2 g once daily was deemed the more appropriate and optimal treatment, despite the effectiveness of penicillin plus gentamicin. The duration of treatment for the patient was determined based on the first negative blood culture after undergoing triple valve heart surgery. The decision to use ceftriaxone over the synergistic penicillin plus gentamicin was based on the patient's relatively stable condition, absence of fever or leukocytosis on presentation, and overall health status. In situations where a patient is allergic to cephalosporins or penicillins, vancomycin can serve as an alternative treatment for four weeks, but close monitoring of renal function is warranted. Ceftriaxone, on the other hand, only requires once-daily intravenous administration and does not necessitate renal dose adjustments or close therapeutic drug monitoring, making it a more optimal choice for this patient [2].

VHD is one of the leading causes of cardiovascular morbidity and mortality globally, with a predominant age-dependence although it can also be congenital. VHD is defined as a cardiac dysfunction caused by structural or functional abnormalities of the heart valves, with the mitral and aortic valves being the most commonly affected [11]. Rheumatic heart disease is the most prevalent VHD globally, but its underreporting is significant due to limited access to diagnostic imaging such as echocardiography in the developing world. In the United States, non-rheumatic VHD is responsible for a higher number of deaths than rheumatic VHD, with 3,046 reported deaths attributed to the latter and 24,811 to the former in 2017 [12].

The four heart valves (mitral, tricuspid, aortic, and pulmonary) are responsible for regulating normal blood flow between the four chambers of the heart and maintaining hemodynamic circulation by controlling pressure gradients. Valve regurgitation results in the backflow of blood into the originating chamber, while valve stenosis creates difficulty in the forward movement of blood. Over time, these pressure differentials can cause cardiac remodeling of the left ventricle through cardiomyocyte hypertrophy [13].

AR can occur as either an acute or chronic condition. Acute AR is characterized by the backward flow of blood from the aorta into the left ventricle during diastole and can result from aortic dissections, infectious endocarditis, or trauma or may be misdiagnosed as other conditions such as sepsis, non-VHD, or pneumonia [14]. In its acute form, AR can rapidly progress to acute heart failure and cardiogenic shock due to hemodynamic collapse. On the other hand, chronic AR is often secondary to hypertension, bicuspid aortic valve, primary aortic disease, rheumatic disease, or calcific degeneration, leading to an increasing left ventricular dilation and eccentric hypertrophy. Although patients may tolerate chronic AR for years, symptoms such as shortness of breath, fatigue, and angina may eventually develop slowly. Surgical aortic valve replacement (SAVR) with either a mechanical or biological prosthesis is the preferred management option when patients become symptomatic or hemodynamically unstable, regardless of left ventricular systolic function. In individuals deemed intermediate or high risk for SAVR, transcatheter aortic valve replacement can be considered. Medical management with ACE inhibitors/ARBs or beta-blockers may be considered if surgical intervention is not feasible [15].

More than two million people in the United States are affected by MR. The condition can be categorized as ischemic or degenerative in developed countries, while it is categorized as rheumatic in developing countries [16]. Chronic MR can present as primary or secondary. Primary MR is a dysfunction of the various parts that make up the valve system, such as the leaflets, annulus, chordae, papillary muscles, or left ventricle free walls, which permit backflow, leading to left ventricular volume overload. The most common causes of primary MR are mitral valve prolapse, rheumatic disease, or radiation therapy. In secondary MR, the valves are usually normal, and the left ventricle is damaged by myocardial infarction, coronary artery disease, or dilated cardiomyopathy, leading to annular dilatation and papillary muscle displacement that causes the valves to leak [17]. MR can manifest as symptoms of heart failure, such as volume overload due to left ventricular dilation and left atrial hypertension, which can progress to pulmonary hypertension and subsequent right ventricular failure. Acute MR can be caused by IE, trauma, such as blunt force, papillary muscle ischemia or rupture, or degenerative disease of the chordae tendinae or leaflets. The treatment for MR includes medical and surgical interventions. Medical therapy involves the use of vasodilators and diuretics to increase forward flow while reducing regurgitant flow and beta-blocker therapy to reverse left ventricular dysfunction. Surgical intervention, specifically mitral valve repair, is the preferred choice in non-rheumatic valves rather than mitral valve replacement [17].

TR occurs when the tricuspid valve in the right side of the heart fails to close properly, causing blood to flow back into the right atrium. This can be a primary condition or can result from left-side heart valve dysfunction, such as mitral and aortic valve diseases, leading to increased pressure in the right side of the heart and regurgitation of blood through the tricuspid valve [18]. In the United States, about 1.6 million people have moderate to severe TR, with its prevalence being even higher in individuals with concomitant VHD or cardiomyopathy [19]. TR is classified as mild, moderate, or severe based on the degree of backflow of blood from the right ventricle into the right atrium. Mild TR may be considered normal if the tricuspid valve structure is normal, but moderate or severe TR is typically caused by leaflet dilatation or abnormalities. Congenital abnormalities, trauma, radiation, pacemaker and defibrillator leads, rheumatic fever, and IE are some of the possible causes of TR. Patients with TR may not experience any symptoms or heart murmurs and, therefore, may be diagnosed incidentally during an echocardiogram. However, if symptoms do occur, they typically present as volume overload.

Medical treatment of TR is conservative through diuretics [20]. This objective can be reached through the repair or replacement of the valve. At this time, there is a preference for tricuspid valve repair, which makes up 89% of tricuspid valve surgeries [20]. The objective of repair is the restoration of leaflet coaptation and correction of annular dilation. Annular dilation is almost always present in severe TR and should be resolved through ring annuloplasty [20].

A study by Kilic et al. demonstrated that in the majority (75.5%) of severe TR cases isolated, annuloplasty was the technique used. Several studies have since shown a decrease in the rate of recurrent TR after the implantation of an annuloplasty ring [20]. In cases where there is significant valvular pathology, replacement is preferred over repair. TR replacement can be obtained through the placement of a mechanical, or if available, bioprosthetic valve. The use of bioprosthetic valves has increased in the United States by 9.2% from 2000 to 2010 [20]. Replacement with a mechanical valve is often preferred in younger individuals with a longer life expectancy, whereas replacement with a bioprosthesis does not require lifelong anticoagulation and is preferred in those with a limited life expectancy, or where the risk of valve thrombosis is needed to be further reduced, bioprosthetic valves have a lower flow rate and decreased pressure at rest than mechanical valves [20].

Alkhouli et al. conducted a study that found a higher incidence of in-hospital death (12% vs. 6.9%), permanent pacemaker implantation (33.7% vs. 11.2%), and blood transfusion associated with tricuspid valve replacement compared to repair [19]. Moreover, a recent comprehensive meta-analysis revealed that tricuspid valve replacement was associated with increased odds of all-cause mortality and a 74% higher risk of postoperative mortality compared to repair. However, it is worth noting that tricuspid valve repair was associated with a higher rate of acute kidney injury [19]. Therefore, careful consideration and individualized treatment planning are necessary for optimal management of TR.

Our patient's case presented a significant challenge. Not only did he have regurgitation in three of his heart valves, the aortic, mitral, and tricuspid valves, but we also suspected that his congenital bicuspid aortic valve played a role in the onset of his condition, potentially contributing to the development of AR. Vegetations from the aortic valve had spread to the mitral valve, exacerbating the already complex situation. Compounding the issue was the fact that the patient had left heart disease, which made the tricuspid valve especially vulnerable to damage. Despite the severity of his valve issues, the patient did not present with any of the typical symptoms associated with valve issues. Instead, he was admitted due to a stroke, adding yet another layer of complexity to an already complicated case. It was determined to proceed with a surgical procedure that includes mitral and aortic valve replacements, as well as tricuspid valve annuloplasty, to effectively address the multiple valve-related issues.

## Conclusions

This paper presents a rare and atypical case of IE involving *G. morbillorum* and an undiagnosed bicuspid aortic valve. Despite the patient’s lack of typical symptoms and significant VHD, the development of valve vegetations caused by *G. morbillorum* resulted in severe regurgitation across the aortic, mitral, and tricuspid valves, making this case highly unusual.

The rarity of this pathogen adds to the uniqueness of the case and emphasizes the need to consider uncommon pathogens in the differential diagnosis of IE, particularly in patients with atypical presentations. Timely identification and management are crucial to prevent serious complications such as stroke and valve damage, which can have significant implications for morbidity and mortality.
